# A Novel Model of Chronic Wounds: Importance of Redox Imbalance and Biofilm-Forming Bacteria for Establishment of Chronicity

**DOI:** 10.1371/journal.pone.0109848

**Published:** 2014-10-14

**Authors:** Sandeep Dhall, Danh Do, Monika Garcia, Dayanjan Shanaka Wijesinghe, Angela Brandon, Jane Kim, Antonio Sanchez, Julia Lyubovitsky, Sean Gallagher, Eugene A. Nothnagel, Charles E. Chalfant, Rakesh P. Patel, Neal Schiller, Manuela Martins-Green

**Affiliations:** 1 Departments of Cell Biology and Neuroscience, University of California Riverside, Riverside, California, United States of America; 2 Bioengineering Interdepartmental Graduate Program, University of California Riverside, Riverside, California, United States of America; 3 Division of Biomedical Sciences, University of California Riverside, Riverside, California, United States of America; 4 Department of Botany and Plant Sciences, University of California Riverside, Riverside, California, United States of America; 5 Department of Bioengineering, University of California Riverside, Riverside, California, United States of America; 6 Hunter Holmes McGuire Veterans Administration Medical Center, Richmond, Virginia, United States of America; 7 Department of Biochemistry & Molecular Biology, Virginia Commonwealth University, Richmond, Virginia, United States of America; 8 Virginia Commonwealth University Reanimation Engineering Science Center, Richmond, Virginia, United States of America; 9 The Massey Cancer Center, Richmond, Virginia, United States of America; 10 Department of Pathology, University of Alabama at Birmingham, Birmingham, Alabama, United States of America; 11 Department of Product Technology, UVP, LLC, an Analytik Jena Company, Upland, California, United States of America; Laurentian University, Canada

## Abstract

Chronic wounds have a large impact on health, affecting ∼6.5 M people and costing ∼$25B/year in the US alone [1]. We previously discovered that a genetically modified mouse model displays impaired healing similar to problematic wounds in humans and that sometimes the wounds become chronic. Here we show how and why these impaired wounds become chronic, describe a way whereby we can drive impaired wounds to chronicity at will and propose that the same processes are involved in chronic wound development in humans. We hypothesize that exacerbated levels of oxidative stress are critical for initiation of chronicity. We show that, very early after injury, wounds with impaired healing contain elevated levels of reactive oxygen and nitrogen species and, much like in humans, these levels increase with age. Moreover, the activity of anti-oxidant enzymes is not elevated, leading to buildup of oxidative stress in the wound environment. To induce chronicity, we exacerbated the redox imbalance by further inhibiting the antioxidant enzymes and by infecting the wounds with biofilm-forming bacteria isolated from the chronic wounds that developed naturally in these mice. These wounds do not re-epithelialize, the granulation tissue lacks vascularization and interstitial collagen fibers, they contain an antibiotic-resistant mixed bioflora with biofilm-forming capacity, and they stay open for several weeks. These findings are highly significant because they show for the first time that *chronic wounds* can be generated in an animal model effectively and consistently. The availability of such a model will significantly propel the field forward because it can be used to develop strategies to regain redox balance that may result in inhibition of biofilm formation and result in restoration of healthy wound tissue. Furthermore, the model can lead to the understanding of other fundamental mechanisms of chronic wound development that can potentially lead to novel therapies.

## Introduction

Failure of acute wounds to proceed through the normal regulated repair process results in wounds that have impaired healing and/or become chronic [2], [3]. Diabetic foot ulcers, venous ulcers, and other similar chronic wounds have a large impact on health, currently affecting ∼6.5 M patients and costing ∼$25B/year in the US alone [1]. Although great efforts have been made to switch the course of repair from non-healing wounds to healing wounds, success has been limited. This is primarily due to the pathophysiological complexity of changing an acute wound into a chronic wound and the lack of good animal models.

Injury causes the early generation of reactive oxygen species (ROS) in the presence of vascular membrane-bound nicotinamide-adenine-dinucleotide (NADH)-dependent oxidases (NOXs) that are produced by resident endothelial cells and fibroblasts [4]. ROS are required for defense against invading pathogens and low levels of ROS act as essential mediators of intracellular signaling that leads to proper healing [5], [6]. However, uncontrolled production of ROS early after injury leads to an altered detoxification process caused by reduction in antioxidant production and activity [7]. Studies have provided evidence that non-healing ulcers in humans have high oxidative and nitrosative stress [8]–[10]. Furthermore, tissue hypoxia as well as anaerobic glycolysis, contribute to the production of lactate and its accumulation under inflammatory conditions [11], [12]. Even in well-oxygenated wounds [11], when the number of neutrophils is high [13], lactate and ROS become significantly elevated as a result of aerobic glycolysis – the so-called “Warburg effect” [14]. This environment leads to a stagnant inflammatory phase. If the inflammatory cells are not removed from the wound tissue, they can promote further tissue damage through excessive production of inflammatory cytokines, proteases, and reactive oxygen intermediates, and increased cell death that, together, result in abnormal granulation tissue development and lead to wounds with impaired healing [15]–[17]. 

Nitric oxide (NO) also plays a key role in wound repair [18], [19]. The beneficial effects of NO in wound repair relate to its functions in angiogenesis, inflammation, cell proliferation, matrix deposition, and remodeling. However, high levels of NO produced by inducible nitric oxide synthase (iNOS) produce peroxynitrite (ONOO^−^), a reactive nitrogen species (RNS). ONOO^−^ causes damage to DNA, lipids and proteins which invariably leads to cell apoptosis and/or necrosis depending on its concentration at the injury site [20].

*It is virtually impossible to study the development of chronic wounds in humans*. By the time these wounds appear in the clinic, the initial stage of development is well passed. Therefore, animal models to conduct studies on the genesis of non-healing chronic wounds are needed. We recently showed that a mouse in which the Tumor Necrosis Factor Superfamily Member 14 (TNFSF14/LIGHT) gene has been knocked out (LIGHT^−/−^ mice) has impaired healing and that the wounds heal poorly and show many of the characteristics of impaired wounds in humans [21]. When compared to control, the wounds of LIGHT^−/−^ mice show defects in epithelial-dermal interactions, high degree of inflammation, damaged microvessels with virtually no basement membrane or periendothelial cells, the collagen in the granulation tissue is mostly degraded, matrix metalloproteinases (MMPs) are elevated and tissue inhibitors of metalloproteinase (TIMPs) are down-regulated. In addition, we also found that sometimes the LIGHT^−/−^ wounds become chronic, and when they do, these defects are highly accentuated. In addition, the wounds become heavily infected with *Staphylococcus epidermidis* [21], a gram-positive bacterium frequently found in human chronic wounds [22]. All of these characteristics are very similar to those found in chronic wounds in humans [23]–[25]. Mechanistically, we have shown that LIGHT mediates macrophage cell death induced by vascular endothelial growth factor (VEGF) and that this occurs in a LTβ receptor-dependent manner [26], indicating that LIGHT is involved in the resolution of macrophage-induced inflammation. In addition, LIGHT^−/−^ mice also show increased levels of Forkhead box protein A1 (FOXA1), Cytochrome P450 2E1 (CYP2E1), and Toll-like receptor 6 (TLR6) which are genes involved in oxidative stress [27]–[30]. Furthermore, Aldehyde oxidase 4 (AOX4) is also elevated in these knockout mice. This enzyme leads to the generation of O_2_^−^ that then aids in release of iron from ferritin [31]. Here we show that by manipulating the microenvironment at wounding we can cause the impaired wounds to become chronic 100% of the time and propose that the same processes are involved in chronic wound development in humans. This model provides an opportunity to understand fundamental mechanisms involved in chronic wound development that can potentially lead to identifying diagnostic molecules and to the discovery of novel treatments.

## Results

**Figure 1:**
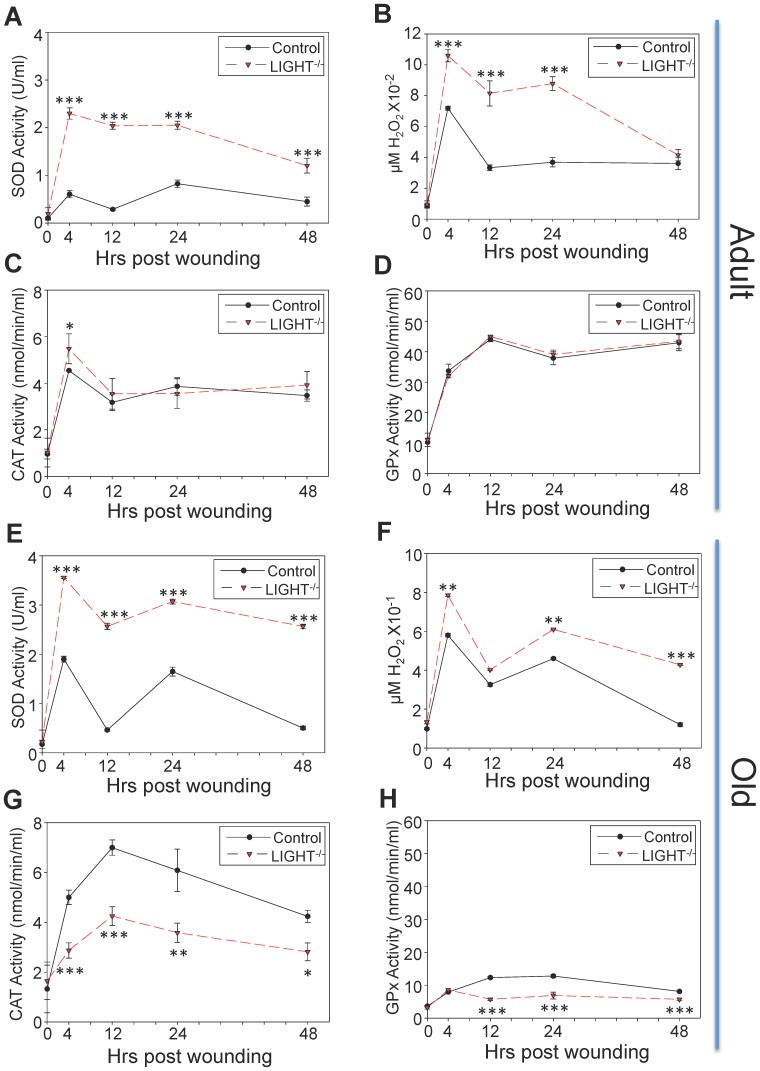
Oxidative stress is elevated in LIGHT^−/−^ wounds. (A) SOD activity was measured using a tetrazolium salt that converts into a formazan dye detectable at 450 nm. SOD activity remains significantly elevated in LIGHT^−/−^ mice in the first 48 hrs post-wounding. *n = 6*. (B) Resofurin formation, detected at 590 nm, was used to determine H_2_O_2_ levels. Significant increases in H_2_O_2_ very shortly post-wounding were seen. *n = 8*. (C) Enzymatic reaction of catalase and methanol in the presence of H_2_O_2_ gives rise to formaldehyde, spectrophotometrically detected with purpald chromogen, at 540 nm. Catalase activity in adult LIGHT^−/−^ and control wounds was similar. *n = 6*. (D) GPx detoxifying activity was measured indirectly at 340 nm by a coupled reaction with glutathione reductase where GPx activity was rate-limiting. The level of GPx activity in the adult LIGHT^−/−^ wounds was essentially identical to that of the controls. *n = 6*. (E-H) The findings in old LIGHT^−/−^ mice were exacerbated in all four parameters when compared to adult LIGHT^−/−^ mice. n = 6. Time zero represents unwounded skin. All data are Mean ± SD. *p<0.05,**p<0.01,***p<0.001.

**Figure 2:**
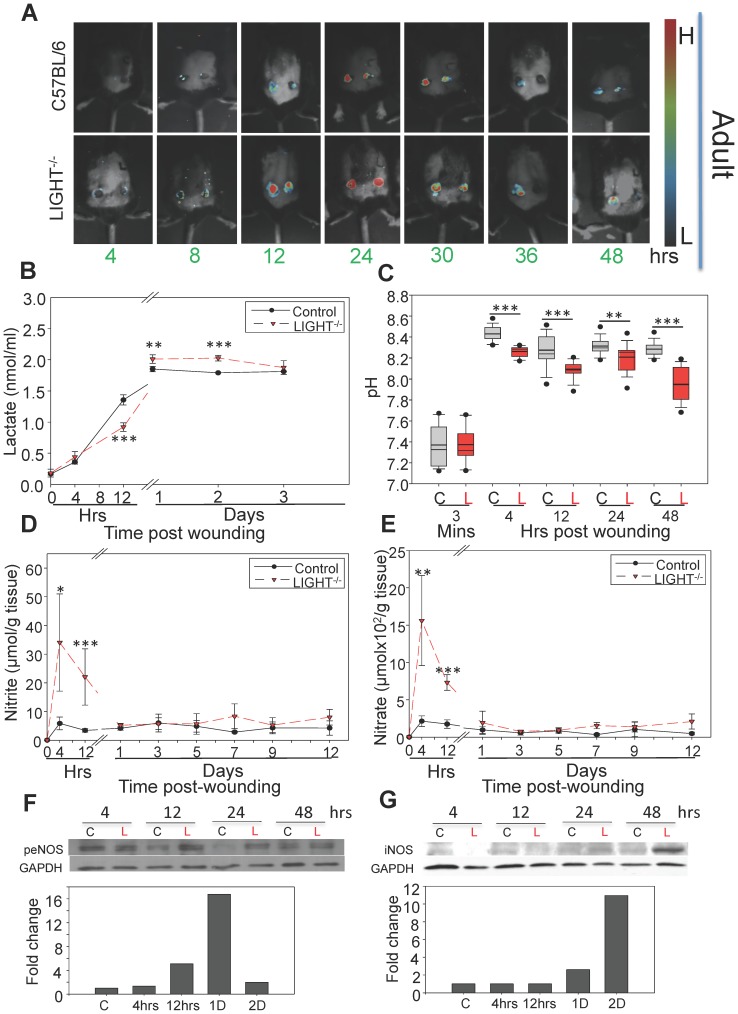
Microscopic, biochemical and chemical markers show imbalanced redox in LIGHT^−/−^ mice. (A) *In vivo* imaging of ROS was carried out using the ImageEM 1K EM-CCD camera with an optical system consisting of a 50 mm f/1.2 lens. Signals were obtained around the periphery of the wound as early as 4 hrs post-wounding in the LIGHT^−/−^ mice and significantly higher signals captured in LIGHT^−/−^ mice peaked at 24 hrs post-wounding. (B) Lactate measurements: An oxidized intermediate was formed when extracted lactate reacted with a probe to give fluorescence detectable at 605 nm. There was significant increase in levels of lactate accumulation in LIGHT^−/−^ mice at 24–48 hrs post wounding. *n = 6*. (C) pH levels were measured using a beetrode microelectrode and micro-reference electrode. The LIGHT^−/−^ wounds were systematically more acidic than controls. *n = 25*. (D,E) Methanolic-extracted nitrite (D) and nitrate (E) were analyzed. Both were greatly increased in LIGHT^−/−^ mice during early response to wounding. *n = 8*. (F-G) Phospho-eNOS levels and iNOS expression in LIGHT^−/−^ wounds were examined by western blotting (representative experiment shown). Analysis by densitometry (normalized to C57BL/6 mouse wound). *Time zero represents unwounded skin except in* *Figure 2C* *. All data are Mean ± SD*. *p<0.05,**p<0.01,***p<0.001.

In order to identify parameters in the wounds with impaired healing that, when changed, may lead these wounds to become chronic, we first characterized the state of ROS/RNS in the early stages of impaired healing by examining a variety of components of the oxidative and nitrosative stress cycle as represented schematically in **Figure S1 in File S1** Superoxide dismutase (SOD) dismutates superoxide anions (O_2_^−^) to generate H_2_O_2_, which can then be detoxified by catalase to H_2_O+O_2_ and by glutathione peroxidase (GPx) to H_2_O. ROS can also enter the Fenton reaction in the presence of ferrous ions to give rise to.OH+OH^−^. O_2_^−^ can also interact with nitric oxide (NO) produced by nitric oxide synthase (NOS) to give rise to peroxinitrite anion (ONOO^−^). The effects of oxidative and nitrosative stress are shown in terms of lipid peroxidation, DNA damage, protein modification and cell death. Secondly, we will present the data on the manipulation of the redox balance that leads to development of chronic wounds including the characterization of the polymicrobial environment that favors growth of biofilm-forming aerobic and anaerobic bacteria. For all figures (**Figures 1** **,** **2** **,** **3** **, and** **4**) except (**Figure 2C**), time t = 0 represents unwounded skin.

**Figure 3:**
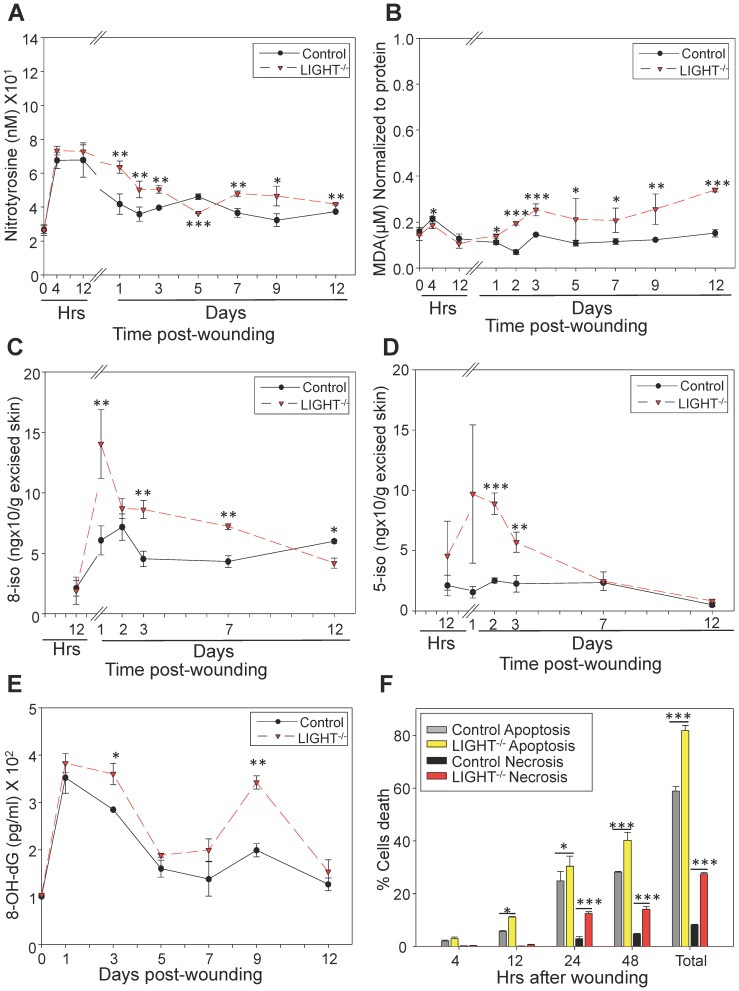
Early oxidative and nitrosative stress in LIGHT^−/−^ wounds have damaging effects on proteins, lipids and DNA and increased cell death. (A) Protein modification measurements were based on a competitive enzyme immunoassay; nitrotyrosine levels in the LIGHT^−/−^ mice were significantly different from control throughout healing. (B) Lipid peroxidation levels were measured fluorometrically at an Ex/Em of 540 nm/590 nm using thiobarbituric acid reactive substances (TBARS); the MDA levels were significantly elevated throughout the course of wound healing in LIGHT^−/−^ mice. *n = 6*. (C, D) F_2_ isoprostanes, were measured using the approach described in the M&M section; levels of 8- and 5-isoprostanes detected in LIGHT^−/−^ mice were much higher than those in the control mice at early times. This correlates with the MDA levels that are the stable byproducts of lipid peroxidation. *n = 5*. (E) Levels of 8-OH-dG, were based on a competitive enzyme immunoassay; the samples were read spectrophotometrically at 412 nm using Ellman's reagent. 8-OH-dG levels were found to be significantly elevated during the course of healing in LIGHT^−/−^ mice. *n = 4*. (F) Cell death by apoptosis and necrosis was determined by staining with Annexin V-FITC and propidium iodide, respectively, followed by FACS analysis. Cell death was increased significantly in the LIGHT^−/−^ mice. The greatest difference occurred with necrosis, which showed to be much higher in LIGHT^−/−^ mice. *Time zero represents unwounded skin. All data are Mean ± SD*. *p<0.05,**p<0.01,***p<0.001.

**Figure 4:**
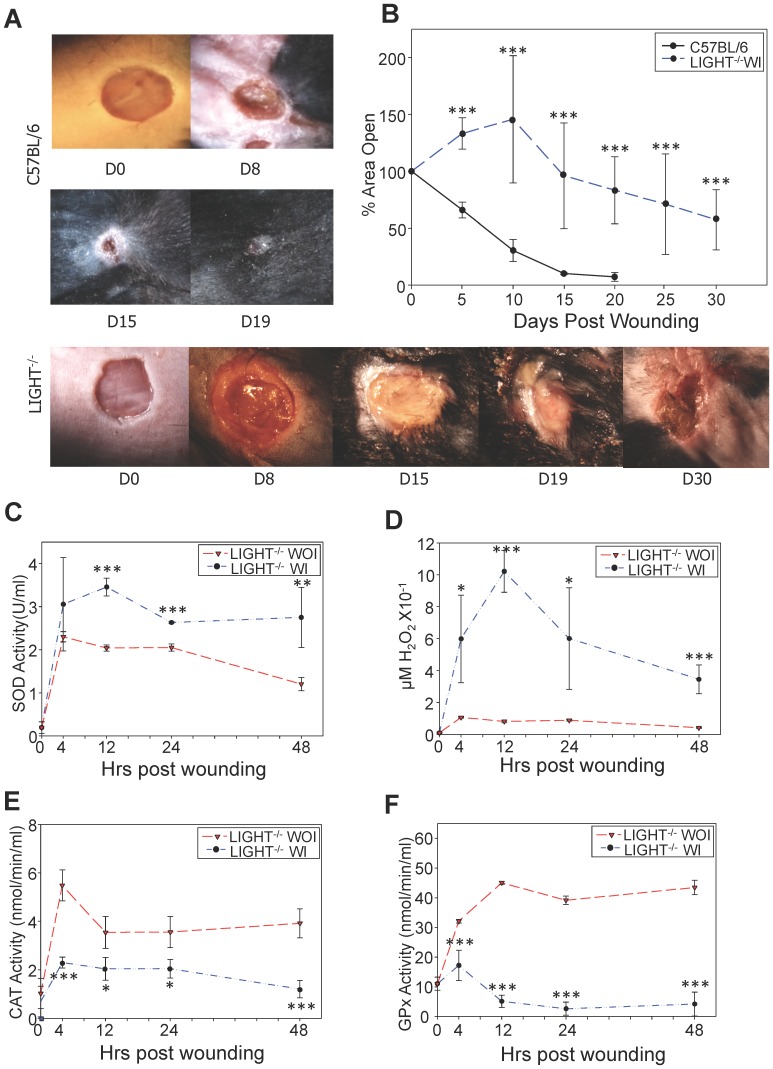
Manipulating redox parameters leads to development of chronic wounds. (A) C57BL/6 and LIGHT^−/−^ mice were wounded and immediately treated with inhibitors for GPx and catalase followed by the application of biofilm-forming bacteria 24 hrs later. The wounds were covered with sterile tegaderm to maintain a moist wound environment and prevent external infection. The LIGHT^−/−^ wounds became chronic and remained open for more than 30 days. *n = 30*. (B) Wound areas were traced using ImageJ and % open wound area was calculated. The LIGHT^−/−^ wounds remained open for significantly longer time than the C57BL/6 wounds with similar treatment. *n = 8*. (C-F) SOD activity (C); H_2_O_2_ levels (D); Catalase activity (E); and GPx Activity (F) were measured as described in Figure 1. All were greatly different from controls. For all tests *n = 6* at minimum. Time zero in C-F represents unwounded skin. All data are Mean ± SD. *p<0.05,**p<0.01,***p<0.001.

### Characterization of the redox environment in wounds with impaired healing *ROS*

#### Oxidative stress

To determine whether the wounds with impaired healing have increased oxidative stress, we measured the levels of SOD. SOD activity was already significantly elevated by 4 hrs post-wounding in the LIGHT^−/−^ wounds compared to the C57BL/6 wounds and remains high through 48 hrs (**Figure 1A**). H_2_O_2_ levels also were significantly elevated as early at 4 hrs post-wounding in the LIGHT^−/−^ wounds, decreasing to control levels by 48 hrs (**Figure 1B**). Furthermore, we observed that in LIGHT^−/−^ mice, both catalase and GPx activities were similar to control mice, suggesting that accumulation of H_2_O_2_ was primarily caused by the inability of the antioxidant system to keep up with the oxidative stress (**Figure 1C,D**).

It is well known that, in human wounds, oxidative stress increases with age. We determined that oxidative stress in wounds of old LIGHT^−/−^ mice also increased with age; higher levels of SOD activity were seen in wounds of old LIGHT^−/−^ mice than in their adult counterparts (**Figure 1E**). H_2_O_2_ levels in the wounds of old LIGHT^−/−^ mice were at least 10 times higher than those in the adult mice (compare **Figure 1F** with **Figure 1B**). In contrast, the level of catalase activity was significantly lower in wounds of old LIGHT^−/−^ mouse than in wounds of old C57BL/6 mice (**Figure 1G**) but was comparable to the wounds of adult LIGHT^−/−^ mice (compare **Figure 1G** with **Figure 1C**). Similarly, GPx activity was significantly lower in the wounds of old LIGHT^−/−^ mice than in old C57BL/6 mice (**Figure 1H**) and was much lower than in either type of adult mice (compare **Figure 1H** with **Figure 1D**). Taken together, these results suggest that adult LIGHT^−/−^ wounds have high levels of oxidative stress and that, much like in humans, these levels are exacerbated with age [24], [32].

To further confirm the elevated presence of ROS we performed real time *in vivo* imaging of excision wounds at various time points after wounding. Imaging was initiated immediately after IP injection of luminol that emits light in the presence of an oxidizing agent such as H_2_O_2_. We detected a signal on the edges of the wound in the LIGHT^−/−^ mouse as early as 4 hrs after wounding. The level of intensity was increased significantly in LIGHT^−/−^ mice compared to C57BL/6 throughout the early hours post-wounding (**Figure 2A**). Similar results were obtained when imaging old LIGHT^−/−^ and C57BL/6 mice (data not shown). These real-time images show for the first time that, *in vivo*, ROS can be detected *in situ* as early as 4 hrs after wounding.

The presence of oxidative stress leads to increase in enzymatic activity of lactate dehydrogenase (LDH), which results in lactate generation. Because ROS-generated oxidative stress is elevated in the LIGHT^−/−^ wounds, we investigated production of lactate in the wound microenvironment [15], [33]. Higher levels of lactate production were seen at 12 hrs post-wounding in the control mice whereas LIGHT^−/−^ mice showed a delayed, but significant, accumulation during days 1 and 2 post-wounding (**Figure 2B**). The levels of lactate accumulation in wounds of old LIGHT^−/−^ mice were similar to the wounds of adult LIGHT^−/−^ mice and also were significantly higher than wounds in old C57BL/6 mice (**Figure S2A in File S1**).

The pH in a wound milieu is a dynamic factor that can change rapidly and affect healing. Studies have shown that the presence of acidic pH correlates with compromised, chronic, and infected wounds [34]. pH measurements of the wound bed were collected immediately, within 3 minutes after wounding and then at the indicated hrs. Relative to the control, the pH obtained from LIGHT^−/−^ wounds was more acidic by 4 hrs post-wounding and remained so through at least 48 hrs (**Figure 2C**). Similar results were obtained with old LIGHT−/− mice (**Figure S2B in File S1**). Unwounded skin surface pH was not measured because the glass microelectrodes we used require moisture and the skin is dry. Humidifying the skin with water will alter the pH because of the presence of free fatty acids on the skin that releases H+ ions into the water applied and can give measurements that are not accurate [35]. Correlations between lactate and pH (proton transport) have previously been shown to increase in parallel to each other [36], [37]. The same occurs in these wounds.

#### Nitrosative stress, protein modification, and damage of lipids and DNA

To determine whether wounds of LIGHT^−/−^ mice have high nitrosative stress, we examined the metabolites of NO, nitrite (NO_2_^−^) and nitrate (NO_3_^−^) and found that shortly after wounding the levels of nitrite in the adult LIGHT^−/−^ mice wounds were very much higher than those in the control at 4 and 12 hrs but declined to normal by day 1 (**Figure 2D**). Nitrate levels showed the same pattern of elevation as nitrite (**Figure 2E**). Old mice showed a similar pattern of elevation but the levels were even higher than in adult LIGHT^−/−^ wounds between 4–12 hrs post-wounding (**Figure S2C,D in File S1**). To determine whether the elevated levels of NO_2_^−^ and NO_3_^−^ early post-wounding were due to changes in nitric oxide synthase (NOS), both endothelial NOS (eNOS) and inducible NOS (iNOS) were examined for phosphorylation/activation of eNOS and elevated expression of iNOS in LIGHT^−/−^ mouse wounds. We found that the levels were significantly elevated but that elevation did not occur until 12 hrs and 24 hrs post-wounding, respectively (**Figure 2F,G**), suggesting that the increase in NO production must be due to activation of other systems/factors occurring very early after wounding.

Modification of tyrosine residues to 3-nitrotyrosine in proteins by ONOO^−^ or other potential nitrating agents occurs when tissues are subject to nitrosative stress. Because we show the presence of nitrosative stress, we examined the levels of 3-nitrotyrosine (3-NT) to assess the effects of this stress on protein modification during healing of the LIGHT^−/−^ mice. We found that the levels of 3-NT were significantly elevated in LIGHT^−/−^ mouse wounds 1 day post-wounding and, except for day 5, remained significantly elevated throughout the course of healing (**Figure 3A**), confirming the deleterious effects of the presence of nitrosative stress. These effects were almost doubled in the old LIGHT^−/−^ mice (**Figure S3A in File S1**).

It is known that increase in ROS/RNS can cause lipid peroxidation. Lipid peroxides are unstable markers of oxidative stress that decompose to form malondialdehyde (MDA) and 4-hydroxynonenal (4-HNE). We found a significant increase in MDA levels 48 hrs after wounding that remained significantly elevated throughout healing (**Figure 3B**). We also found that the levels of lipid peroxidation were exacerbated in wounds of old LIGHT^−/−^ mice after 48 hr post-wounding (**Figure S3B in File S1**). Furthermore, we used mass spectroscopy to examine whether ROS-induced non-enzymatic peroxidation products of arachidonic acid, such as isoprostanes, were present in the wounds. We found that 8-isoprostane (8-epi-PGF_2α_) and 5-isoprostane were significantly elevated in the LIGHT^−/−^ mouse wounds, suggesting the breakdown of arachidonic acid in the presence of ROS (**Figure 3C,D**). These results confirm that there is lipid damage in the LIGHT^−/−^ wounds.

Another detrimental effect caused by excessive oxidative stress and nitrosative stress is 8-hydroxylation of the DNA guanine base (8OHdG) that results in DNA damage. The overall levels of this stress marker were increased in wounds of adult LIGHT^−/−^ mice (**Figure 3E**), with significant increase at days 3 and 9 post-wounding. We also found that the levels of 8-OHdG in the old LIGHT^−/−^ mouse wounds were significantly more elevated throughout the course of healing (**Figure S3C in File S1**).

#### Levels of cell death by both apoptosis and necrosis

Given that excessive redox stress results in damage of DNA, proteins, and lipids that are critical for cell survival and function, we examined cell death both by apoptosis and necrosis (**Figure 3F**). Apoptosis was significantly increased 12 hrs post-wounding and increased even more by 48 hrs post-wounding. Cell death by necrosis was predominantly found at 24 hrs and 48 hrs. Particularly striking is the difference in cell death by necrosis between control and LIGHT^−/−^ mice. This elevated cell death is potentially due to the higher levels of oxidative stress and can lead to chronic inflammation, impaired healing and delayed wound closure.

### Manipulation of redox balance and the presence of biofilm-forming bacteria lead to development of chronic wounds

Our previous results [21] and the results presented above strongly suggest that the LIGHT^−/−^ impaired healing is caused by redox imbalance established shortly after injury, resulting in excessive cell death which then creates an environment that increases inflammation and is propitious for the growth of biofilm-forming bacteria, thereby setting the wound on a course that leads to development of chronic ulcers. To test this possibility we significantly increased the oxidative stress in the wound by further inhibiting the antioxidant enzymatic activity and applying the biofilm-forming bacteria, S. *epidermidis* C2, that we isolated from the spontaneously-developed chronic wounds of the LIGHT^−/−^ mice [21]. Inhibition of catalase by 3-Amino-1,2,4-triazole (ATZ) and GPx by mercaptosuccinic acid (MSA) immediately post-wounding and application of S. *epidermidis* C2 24 hrs later was sufficient to turn the wounds with impaired healing into chronic wounds 100% of the time (**Figure 4A**). Chronic wounds were successfully created in 30 animals used in 10 different experiments. Wounds of C57BL/6 mice treated under the same conditions closed in 15–19 days whereas the LIGHT^−/−^ wounds remained open for>4 weeks (**Figure 4B**). The wounds were kept covered at all times using sterile tegaderm and changed upon compromised sealant of the bandage.

Following the application of inhibitors post-wounding, and bacteria 24 hours later, we evaluated the levels of ROS to determine whether the levels of oxidative stress increased. With antioxidant inhibitor treatment, SOD (**Figure 4C**) and H_2_O_2_ levels (**Figure 4D**) were significantly elevated by 12 hours post-wounding. Corresponding to the increase in ROS, antioxidant enzymes catalase and GPx, that were inhibited by ATZ and MSA respectively, were decreased significantly (**Figure 4E,F**). These experiments were conducted simultaneously, under identical conditions.

**Figure 5:**
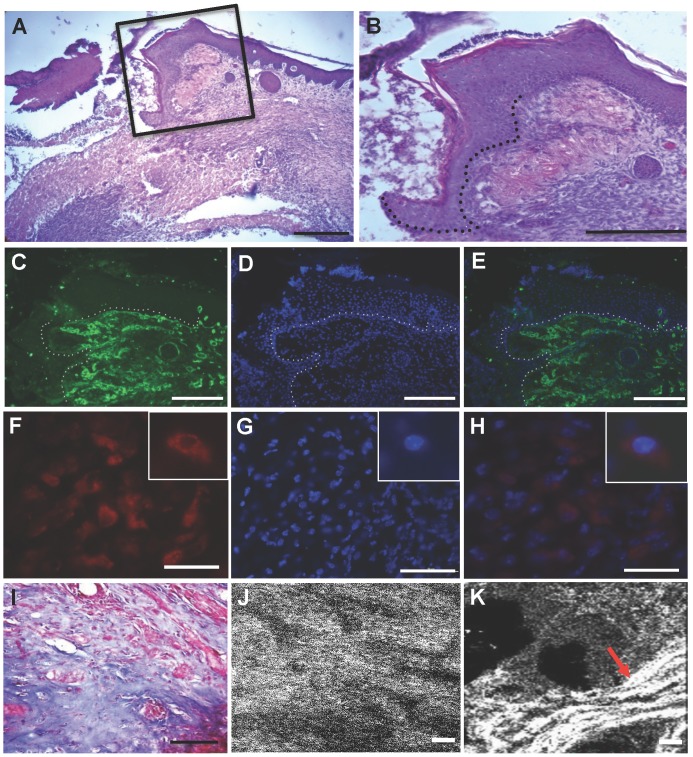
Histological evaluation of chronic wounds. (A) Representative picture of H&E-stained sections of a LIGHT^−/−^ chronic wounds from an animal treated with catalase and GPx inhibitors and the application of bacteria. The epithelium does not cover the wound tissue and the granulation tissue is poorly formed. Scale bar 500 µm. (B) Higher magnification of the boxed area in (A). Epithelial tongue is outlined with a dotted line (compare with Figure S4A). Scale bar 100 µm. (C) Immunolabeling for Collagen IV delineates the presence of basement membrane; dotted line marks where basement membrane is missing in the migrating tongue. (D) propidium iodide staining identifies cell nuclei. (E) Merger of (C) & (D). (F) Immunolabeling for F4/80, a marker for macrophages, to illustrate the presence of inflammation; (G) propidium iodide staining identifies cell nuclei. (H) Merger of (F) & (G). Inserts are high magnifications of a single macrophage. (I) Representative Masson-trichrome (blue color) stained section illustrating loss of collagen bundles; scale bar 100 µm. (J,K) SHIM analysis of a similar section (J) confirms results in (I) and, for comparison, collagen in the granulation tissue of a normal wound similarly analyzed by SHIM (K) showing filamentous collagen (red arrow); scale bar 10 µm.

Histological examination of chronic LIGHT^−/−^ wounds showed that the migrating tongue of the epidermis was blunted and tortuous (**Figure 5A,B**) rather than thin and linear as in the control (**Figure S4A in File S1**). Also, the granulation tissue was poorly developed (**Figure 5A**) when compared to normal granulation tissue (**Figure S4B,C in File S1**). Collagen IV, a component of the basal lamina, was well-formed behind the migrating tongue but was absent under the tortuous migrating edge (**Figure 5C-E**). We also found that these wounds contain macrophages, indicating that inflammation has not been resolved (**Figure 5F,H**; inserts show higher magnification of one macrophage). Furthermore, the interstitial collagen deposition and organization were abnormal in the LIGHT^−/−^ chronic wounds as revealed by Masson trichrome staining (**Figure 5I**) and by second harmonic generation imaging microscopy (SHIM) (**Figure 5J,K**). Although interstitial collagen was present, the collagen fibers were not clearly visible and did not form proper bundles (**Figure 5J**). This is similar to the finding we published on the impaired wounds of LIGHT−/− wounds [21] but much more exaggerated.

To determine whether the application of the bacteria alone or in the presence of a single inhibitor could induce chronicity in the LIGHT^−/−^ wounds, we introduced S. *epidermidis* C2 24 hrs post-wounding without any inhibitor treatment (**Figure S5A in File S1**) or with just ATZ treatment (**Figure S5B in File S1**) or with just MSA treatment (**Figure S5C in File S1**). In all three cases, both in the C57BL/6 and LIGHT^−/−^ mice, the wounds healed by day 15–19, suggesting that development of chronic wounds requires all three of these elements: inhibition of both catalase and GPx to greatly decrease the antioxidant enzymes in the wound, plus addition of biofilm-forming bacteria.

**Figure 6:**
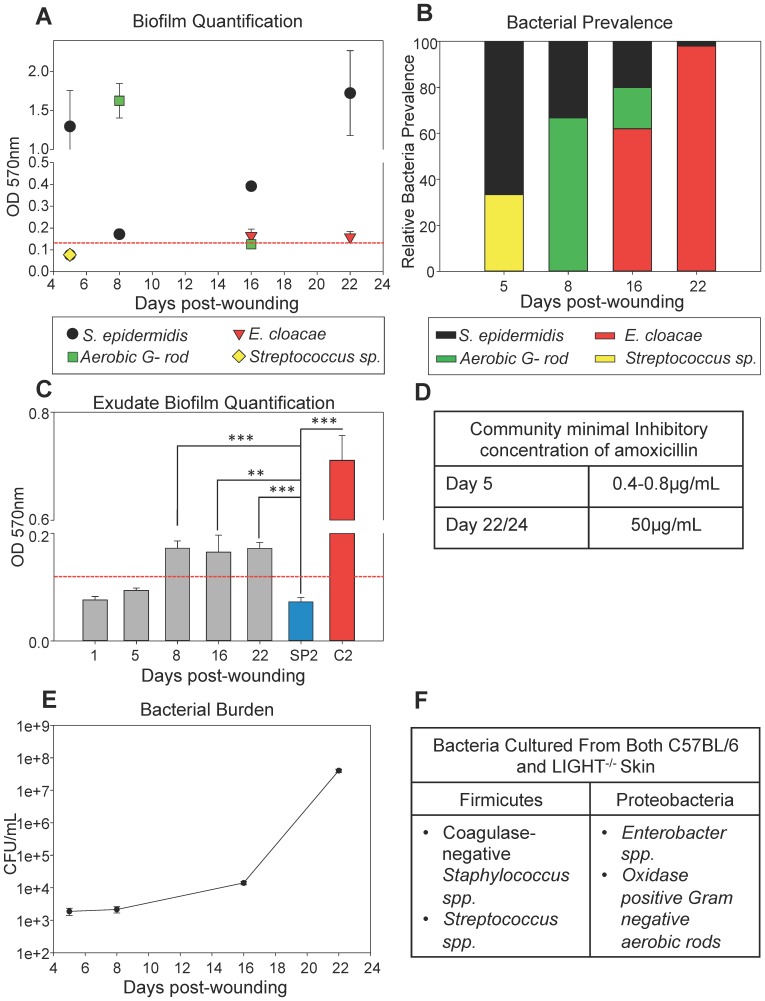
Identification and characterization of the microflora that colonizes the LIGHT^−/−^ chronic wounds. (A) Biofilm production was quantified by measuring the optical densities of stained bacterial films adherent to plastic tissue culture plates. Biofilm forming capacity of *S. epidermidis* was seen throughout the time course of chronic wounds. *n = 7*. (B) Bacterial identification was carried out by growing bacteria on tryptic soy agar. Gram-negative rods were characterized using the API 20E identification kit. *n = 7*. (C) Biofilm quantification of exudate obtained from wounds was performed at OD570 nm. The dynamics of the polymicrobial community in the wounds does not seem to affect the overall degree of biofilm production during the later stages of healing. Controls used were biofilm-negative (OD570 nm<0.125) *S. hominis* SP2 and biofilm-positive *S. epidermidis* C2. *n = 8*. (D) Antibiotic challenge on wound exudates collected from LIGHT^−/−^ mice was done using Amoxicillin. The CMIC of amoxicillin on the bacteria found in the chronic LIGHT^−/−^ wound exudate at day 22/24 was 50 µg/ml, much higher than exudate collected at day 5 when biofilm is not yet abundant. (E) Bacterial burden was evaluated by colony forming unit counts. The CFU/mL was relatively low during the early phases of healing and was highest during the impaired and chronic stages of healing. *n = 7*. (F) Normal skin swabs were collected from LIGHT and C57BL/6 mice to evaluate resident organisms. The microbiota of the skin was similar in both C57BL/6 and LIGHT^−/−^ mice.

It has been established that the bioflora that colonize chronic wounds in humans is commonly polymicrobial [38], [39]. Therefore, we determined whether the LIGHT^−/−^ chronic wounds also exhibited this polymicrobial phenotype. Wound exudates from both adult and old LIGHT^−/−^ mice were collected and the bacteria genus/species determined as described in Materials and Methods. In addition, staining of adherent cells with Hucker crystal violet, which has been widely used as readout for biofilm-production [40]–[43], was used as a qualitative measure for biofilm formation in our bacteria isolates. As expected, we found that biofilm-forming (OD570 nm≥0.125) coagulase-negative *Staphylococcus epidermidis* was present in the wounds throughout healing, given that we infected the wounds with *S. epidermidis* C2. However, co-colonizing bacteria were also isolated. These co-colonizers were identified as non-biofilm forming hemolytic *Streptococcus sp.*, biofilm-producing oxidase-positive aerobic Gram-negative rods (presumptively *Pseudomonas*), and *Enterobacter cloacae* (dotted line in **Figure 6A** defines the minimum optical density for biofilm formation).

Quantification of the relative bacterial prevalence showed that the dynamics of the colonizing bioflora in adult LIGHT^−/−^ mouse wounds changes over time (**Figure 6B**). These changes are marked by the decreased concentration of *S. epidermidis* populations coupled with the appearance of the oxidase positive Gram-negative rods followed by *E. cloacae*. As the wound progresses to a non-healing/chronic stage at ∼20 days post-wounding, the *E. cloacae* population dominates the wound with traces of *S. epidermidis* (**Figure 6B**). Irrespective of the shift in bacterial population of the wounds, the overall degree in biofilm production by these polymicrobial communities (dotted line in **Figure 6C**) did not change significantly over time at least until 22 days. However, the individual contribution to biofilm production varies and is dependent on the time of isolation and is species specific (**Figure 6C**). Eight days post-wounding, biofilm-producing *Staphylococcus epidermidis* (C2) is significantly different from the non-biofilm-producing negative control, *Staphylococcus hominis* (SP2, ATCC 35982). SP2 does not adhere to polystyrene plates, does not produce extracellular polysaccharide and is a commensal bacterium found on human skin [44]. Because of these characteristics, this strain has been widely used as a negative control for biofilm production [40], [45], [46]. Similar observations were made in the old LIGHT^−/−^ mice (**Figure S6A-C in File S1**).

It has been well established that biofilm-associated wound infections are extremely resistant to antimicrobial therapy [47], [48]. The community minimal inhibitory concentration (CMIC) of amoxicillin required to inhibit the growth of biofilm-producing microbial flora from LIGHT^−/−^ adult chronic wounds was determined to be 50 µg/mL (day 22/24) compared to the 0.4–0.8 µg/mL required for non-biofilm producing colonizers (day 5) (**Figure 6D**). This suggests that biofilm-producing microbial flora isolated from LIGHT^−/−^ chronic wounds are ∼50X more resistant to killing by amoxicillin compared to their non-biofilm producing counterparts.

It has been reported that the majority of chronic wounds in humans have bacterial contamination and high levels of bacterial burden will likely result in impaired healing [49]. At 5 and 8 days post-wounding, colony-forming unit counts (CFU/mL of exudate) from adult and old LIGHT^−/−^ mouse exudates show low levels of bacterial burden (1.6×10^3^ CFU/mL and 2.0×10^3^ CFU/mL respectively). However, these levels reach 4.0×10^7^ CFU/mL and 7.4×10^7^ CFU/mL by 22–24 days of healing (**Figure 6E** **and Figure S6D**).

In order to determine whether the skin of mice contain the bacteria that eventually make biofilm in the chronic wounds, we took skin swabs from unwounded C57 and LIGHT^−/−^ mice and cultured them *in vitro* (**Figure 6F**). The majority of the cultured bacteria belong to the Firmicutes phylum, specifically *Staphylococcus spp. and Streptococcus spp.* We also documented the presence of bacteria that belong to the Proteobacteria phylum (e.g. various Gram-negative rods and *Enterobacter*). These bacteria are all known to be associated with the human skin microbiota [50].

**Figure 7:**
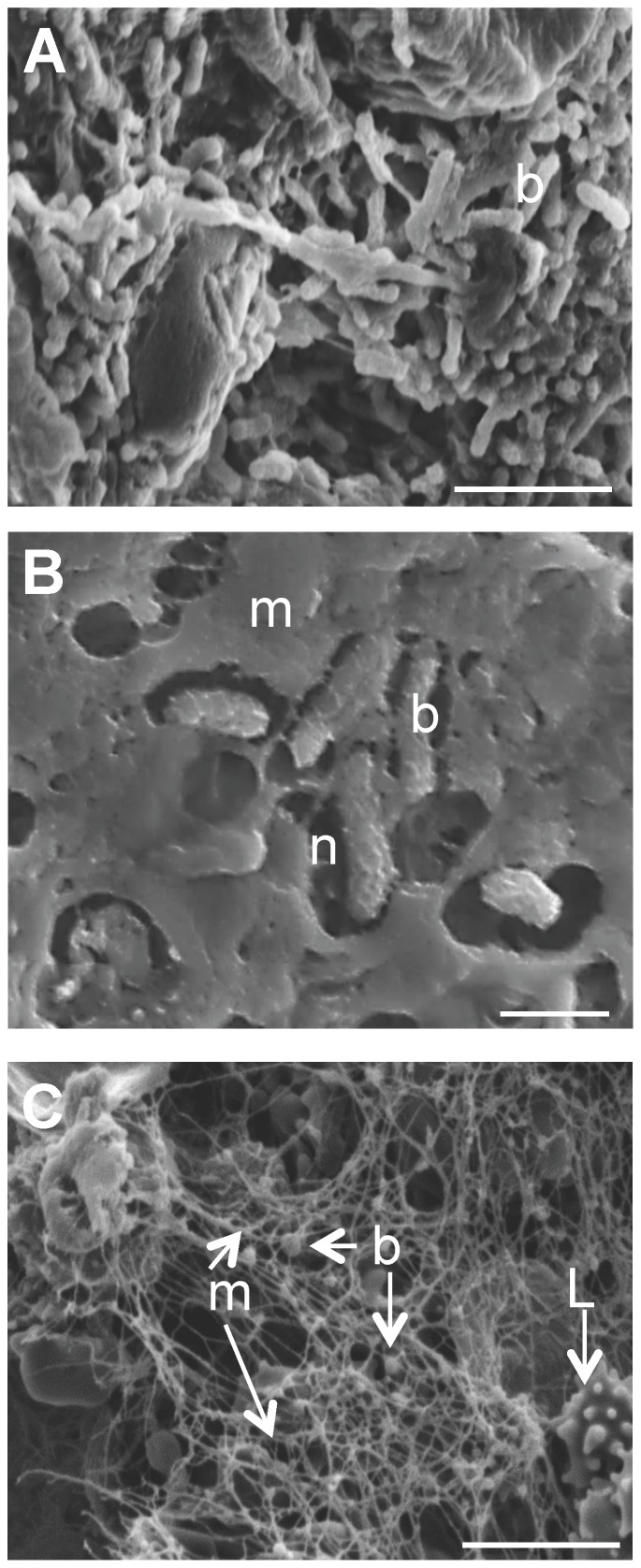
Morphological characterization of biofilm present in LIGHT^−/−^ wounds. Scanning electron microscopy (SEM) images of the Au/Pd sputtered, fixed and dried, chronic wound samples were captured using an FEI XL30 FEG SEM. (A) Image shows the presence of bacterial rods (b) in the wound bed. (B) High magnification image of bacteria embedded in a biofilm-associated matrix (m) in a well-defined niche (n). (C) Matrix beneath the biofilm showing the presence of matrix (m) and of cocci bacteria (b). A Lymphocyte (L/arrow) was highlighted for size references. Scale bars 5 µm (A,C) and 1 µm (B).

To further confirm the presence of biofilm-forming bacteria in these wounds we performed scanning electron microscopy on LIGHT−/− chronic wounds. An abundance of bacteria was observed in the wound and some of those bacteria were embedded in a biofilm-like matrix (**Figure 7A**), with some of them appearing to reside in a defined niche surrounded by matrix (**Figure 7B**). Beneath the biofilm we observed the presence of numerous inflammatory cells adherent to extracellular matrix (**Figure 7C**). Furthermore, analysis of the glycosyl composition of the exudate collected from the chronic wounds showed high levels of N-acetylglucosaminyl (GlcNAc), galacturonosyl (GalU), mannosyl, galactosyl and glucosyl residues (data not shown). This glycosyl composition is consistent with the presence of extracellular polysaccharide material, and possibly N-glycoproteins, in the chronic wound. These carbohydrates have also been shown to be present in human chronic wounds during *P. aeruginosa* infections [51] and more recently exopolysaccharides with glycosyl compositions including these residues have been characterized in other species such as *Staphylcococcus* and *Enterobacter* which are pathogens commonly found in humans [52].

## Discussion

We have shown that we can *create chronic wounds* by manipulation of the impaired wounds of LIGHT^−/−^ mice using antioxidant enzyme inhibitors to further increase ROS/RNS and by adding biofilm-forming bacteria previously isolated from the naturally occurring chronic wounds of these transgenic mice. This approach leads to the generation of chronic wounds 100% of the time. These wounds: (1) Contain high levels of reactive oxygen and nitrogen species and, much like in humans, these levels increase with age; (2) have decreased levels of anti-oxidant enzymes indicating the buildup of oxidative stress in the wound environment; (3) contain increased peroxynitrite and lipid peroxidation-derived products, increased 3-nitrotyrosine levels, increased DNA damage and high levels of cell death, contributing to redox imbalance in the wound microenvironment; (4) do not heal for weeks.

Our data show that SOD enzymatic activity is highly elevated in the first 48 hrs post-wounding which likely is the cause for continued increase of H_2_O_2_ at the wound site. This is particularly important because the activities of the antioxidant enzymes, catalase and GPx, are not elevated to compensate for the extra H_2_O_2_ produced. In old animals, catalase and GPx activity is even lower than in the control, exacerbating the levels of H_2_O_2_. Furthermore, not only can H_2_O_2_ cause damage directly, it can also enter the Fenton reaction in the presence of divalent iron ions to produce hydroxyl radicals (.OH) that lead to additional tissue damage [8], [53], [54]. 

The LIGHT^−/−^ wounds also have high levels of inflammatory cells early after wounding that persist for a long time [21]. Increase in inflammation in a hypoxic wound tends to drive lactate accumulation that, in turn, leads to an unchecked proton gradient. As a consequence, lactate plays an important role in maintaining the fine acid-base milieu [11]–[13]. LIGHT^−/−^ wounds showed increases in lactate levels both in adult and old mice, suggesting a pH imbalance. Recent findings on successful acceptance of skin grafts on chronic wounds was higher at elevated pH (alkaline) than at lower (acidic) pH [36], [55], [56]. In the control wounds, the pH shifted to alkaline at 4 hrs whereas in the LIGHT^−/−^ wounds it shifted to more acidic and the levels remained acidic throughout at least the first 2 days in both adult and old LIGHT^−/−^ mice, potentially contributing to the impaired healing in the wounds of these mice. Although we do not know whether increases in anaerobic metabolism are due to the down regulation of oxidative phosphorylation in an effort to alleviate oxidative stress, we are currently studying the gene expression profiles of the LIGHT^−/−^ wound very early post wounding to obtain in-depth insight into the genes/proteins responsible for such processes.

The levels of nitrite and nitrate, end products of NO metabolism, were significantly elevated very early post-wounding in the adult and old LIGHT^−/−^ mice. This suggests excessive levels of NO production at the wound site that in the presence of O_2_^−^ can generate ONOO^−^. It has been reported that phosphorylation of eNOS modulates both the production of NO and O_2_^−^ [57] and also that increase in H_2_O_2_ may exert effects on endothelial cell dysfunction and uncoupling of NOS. Our data show that there is increased phosphorylation/activation of eNOS and increased iNOS levels. However, the elevated levels of phospho-eNOS and of iNOS appear after the increase in nitrite and nitrate levels in LIGHT^−/−^ wounds, hence these enzymes cannot be the reason for the increases in nitrite and nitrate. It is possible that elevation of NO could be the result of either dephosphorylation of Thr495 on eNOS [58] or increases in L-arginine [59] and decrease in endogenous NOS inhibitors [60]. Furthermore, the elevation in eNOS and iNOS at later times after wounding suggests an increase in NO that can combine with O_2_^−^ to give rise to ONOO^−^, a highly damaging ion species.

Clinical studies on chronic wounds in humans have shown free-radical-induced damage of proteins, lipids and DNA [18], [20], [61], [62]. We found that the levels of malondialdehyde (MDA), a byproduct of lipid peroxidation, were significantly elevated throughout the course of healing in LIGHT^−/−^ mice, indicating lipid damage. We also show the presence of F_2_ isoprostanes that are considered to be the gold standard of oxidative stress and lipid peroxidation. Levels of 8- and 5-isoprostanes detected in LIGHT^−/−^ mice were significantly elevated when compared to the control mice.

DNA damage induced by ROS and RNS can cause modifications that impair DNA repair [63]. Levels of excretion of the free, water soluble, 8-OHdG are reduced, resulting in failing exonuclease activity; this is especially seen with aging, leading to cell damage [64], [65]. Our results show two waves of DNA damage in LIGHT^−/−^ mouse wounds. The first wave suggests that the initial accumulation of 8-OHdG in these wounds is potentially controlled by existing exonuclease activity. However, once this activity is exhausted, the cells are no longer able to handle DNA damage. Increased levels of DNA damage in old mice are seen early and remain elevated, underscoring the continuous increase in oxidative and nitrosative stress with aging of this mouse model much as is seen in humans.

NO is a dynamic molecule that reacts with O_2_^−^ at a rate constant three times higher than the rate constant reaction of O_2_^−^ with SOD, giving rise to ONOO^−^ production [66] that causes nitration of tyrosine residues which damages proteins. Previous studies have shown that changes on tyrosine residues in proteins is an irreversible process that, in turn, severely impairs the regulatory components that undergo phosphorylation or adenylation in signal transduction events [67]. The increased levels of nitrotyrosine in adult and exacerbation in old LIGHT^−/−^ mice suggest an increase in tissue damage.

NO plays a crucial role in bacterial biofilm dispersion [68]. Recent studies have shown that the use of low doses of NO in conjunction with antibiotics can lead to bacterial biofilm dispersion and induce these biofilm-forming bacteria to behave in a planktonic manner, hence reducing biofilm formation [69]–[71]. Although NO also has been widely considered an important immune cell regulator and a chemoattractant that plays a vital role in signaling events in wound healing [72], the chemistry of excessive NO changes in the presence of oxygen. These two molecules readily react with each other, giving rise to RNS that cause damage in the wound tissue [73], [74]. Formation of ONOO^−^, triggered by combination of NO and O_2_^−^, has been known to cause nitrosative damage. We show that there are elevated levels of nitrosative damage in LIGHT^−/−^ wounds after we increase the oxidative stress in the wounds. Therefore, we speculate that the complexity of the microbiota and varying levels of NO, due to the presence of ROS, can lead to restricted levels of biofilm dispersion.

Occurrence of oxidative stress and maintenance of redox balance following stress is an essential component for proper wound healing. Stress is triggered by increases in ROS produced by (i) inflammatory cells (termed as oxidative burst), (ii) a family of NADPH oxidase (NOX), consisting of NOX1-5, and DUOX1&2, [73] and (iii) wound fibroblasts when stimulated by pro-inflammatory cytokines [75]. Although moderate increases in ROS regulate various signaling processes and act against invading bacteria, prolonged and excessive presence of ROS and inflammation can lead to hypoxia and tissue damage caused primarily by lipid peroxidation, DNA damage, protein nitrosylation, and cell death. Furthermore, excessive levels of ROS due to tissue damage create an environment that can serve as an inviting substrate for bacteria to thrive upon. The relationship between exacerbated levels of ROS and bacteria has been reported previously [76], [77]. Studies have suggested that DNA double-stranded breaks in bacteria caused by oxidative stress lead to mutagenic repair via DNA repair protein RecA, rendering the variants with increased antibiotic resistance and adaptability to the surrounding microenvironment [76]. Furthermore, during excessive oxidative stress, these bacteria upregulate genes that increase their virulence [77]. In an effort to obtain excessive and persistent levels of oxidative stress, we manipulated the early wound microenvironment by inhibiting catalase [78] and GPx activities [79] and introduced our previously isolated S. epidermidis bacterial strain. The intensified levels in redox stress and the presence of biofilm-forming bacteria led to reproducible generation of chronic wounds.

Chronic wounds, and difficult-to-heal wounds are postulated to have an underlying biofilm-associated microbial contribution that is complex and dynamic [23], [49], [80], [81]. We show that chronicity in LIGHT^−/−^ wounds is accompanied by a persistent bacterial infection that is polymicrobial and contains biofilm-producing bacteria. The colonizing bacterial species associated and/or responsible for the formation of chronic wounds in the LIGHT^−/−^ mice, are biofilm-producing and the capacity of these organisms to produce biofilms varies depending on the time of isolation. Furthermore, we showed that the source of infection arises from the LIGHT^−/−^ mouse skin microbiota. Similar observations have been documented for human chronic wounds [82], [83].

The presence of biofilm-producing *S. epidermidis* in human chronic wounds [23] and the contribution of *E. cloacae* in nosocomial infections are well known [84]. However, much less is known about *E. cloacae* infection in chronic wounds, although its presence is often reported [85] in diabetic foot infection [86], [87], diabetic gangrene [88], and chronic venous leg ulcers [89], [90]. Perhaps the lack of consideration of *E. cloacae* contribution to the development of chronic wounds may be in part due to their relatively lower initial abundance compared to the more commonly isolated bacteria *Staphylococcus spp.*, *Enterococcus spp.*, and *Pseudomonas aeruginosa* [49], [90], [91].

## Summary and Conclusions

The chronic wound model we present in this publication is the first to effectively mimic chronic wounds in humans. The model wounds stay open for weeks and capture many of the characteristics of human chronic wounds. LIGHT^−/−^ mice have elevated levels of genes involved in oxidative and nitrosative stress that lead to imbalanced redox levels in the wound tissue that are exacerbated with age. As a consequence, redox burden causes deleterious effects in the wound tissue that lead to impaired healing. Manipulation of the wound microenvironment to increase oxidative stress in the presence of biofilm-forming bacteria leads inevitably to development of chronic wounds, identifying high levels of oxidative stress in the wound tissue as a critical factor for chronic wound development. Furthermore, because of the nature and complexity of the mixed wound microbiota, the model presented here can provide insight into the biology of bacterial dynamics and host interaction and the factors that promote biofilm production. This model system, in which a genetic alteration leads to an imbalance in redox levels in wound tissue, has the potential to lead to the understanding of other fundamental cell and molecular mechanisms of chronic wound development and, by implication, to the development of new therapies. Having established a system where 100% of wounds are chronic, we are currently working on reversing chronicity in LIGHT^−/−^ and other genetically-modified mice.

## Materials and Methods

### Dermal excisional wound model

Animals were housed at the University of California, Riverside (UCR) vivarium. All experimental protocols were approved by the UCR Institutional Animal Care and Use Committee (IACUC). Experiments were performed using 12–16 week old mice categorized as adult mice and 85–92 week old mice as old mice. The procedure used was performed as previously described [21].

### Superoxide dismutase activity assay

Total tissue superoxide dismutase (SOD) activity was measured by using a commercially available kit (Cayman Chemical, Catalog# 706002, Ann Arbor, USA) that measures all three types of SOD (Cu/Zn-, Mn-, and EC-SOD). One unit of SOD is defined as the amount of enzyme needed to cause 50% dismutation of the superoxide radical. Extracts obtained from tissues collected at 4 hr, 12 hr, 24 hr and 48 hr post-wounding were processed for total SOD activity according to the protocol provided by the assay kit manufacturer. The SOD activities of the samples were calculated from the linear regression of a standard curve that was determined using the SOD activity of bovine erythrocytes at various concentrations run under the same conditions. The SOD activity was expressed as U/ml of tissue extract.

### Hydrogen peroxide activity assay

Tissue hydrogen peroxide (H_2_O_2_) levels were measured by using a commercially available kit (Cell Technology Inc., Catalog# FLOH 100-3, Mountain View, USA) that utilizes a non-fluorescent detection reagent. The assay is based on the peroxidase-catalyzed oxidation by H_2_O_2_ of the nonfluorescent substrate 10-acetyl-3,7-dihydroxyphenoxazine to a fluorescent resorufin. Fluorescent intensities were measured at 530 nm (excitation)/590 nm (emission) using a Victor 2 microplate reader. The amounts of H_2_O_2_ in the supernatants were derived from a seven-point standard curve generated with known concentrations of H_2_O_2_.

### Catalase activity assay

Tissue catalase activity was measured by using a commercially available kit (Cayman Chemical, Catalog# 707002, Ann Arbor, USA). The enzyme assay for catalase is based on the peroxidatic function of catalase with methanol to produce formaldehyde in the presence of an optimal concentration of H_2_O_2_. The formaldehyde produced was measured spectrophotometrically, with 4-amino-3-hydrazino-5-mercapto-1,2,4-triazole (purpald) as the chromogen, at 540 nm in a 96-well place. The catalase activity was expressed as nmol/min/ml of tissue extract.

### Glutathione peroxidase activity assay

Tissue glutathione peroxidase (GPx) activity was measured using a commercially available kit (Cayman Chemical, Catalog# 703102, Ann Arbor, USA). The activity was measured indirectly by a coupled reaction with glutathione reductase (GR). GPx reduces H_2_O_2_ to H_2_O and in the process oxidized glutathione (GSSG) is produced that in turn is recycled to its reduced state by GR and NADPH. Furthermore, oxidation of NADPH to NADP^+^ is accompanied by a decrease in absorbance at 340 nm. Under conditions in which GPx activity is rate limiting, the rate of decrease in the absorbance measured at 340 nm, in a 96-well plate at 1-min interval for a total of 5 min using a Victor 2 microplate reader, is directly proportional to the GPx activity of the sample. GPx activity was expressed as nmol/min/ml of tissue extract.

### Lactate measurement assay

Tissue lactate levels were measured using a commercially available kit (Biovision Inc, Catalog# K638-100, Milipitas, USA). Tissue lactate extracts were specifically oxidized to form an intermediate that reacts with a colorless probe to generate fluorescence that was measured at 530 nm (excitation)/590 nm (emission) using Victor 2 microplate reader. The intensity was directly proportional to the amount of lactate measured in nmol/ml.

### pH measurements

Wound pH levels were measured using a Beetrode micro pH electrode with a 100 µm tip diameter, 2 mm receptacle (World Precision Instruments, Catalog# NMPH5, Sarasota, USA). A separate reference electrode of 450 µm diameter tip was used (World Precision Instruments, Catalog# DRIREF-450, Sarasota, USA) along with a small, battery-operated compensator (World Precision Instruments, Catalog# SYS-Beecal, Sarasota, USA) to generate mV readings in the range of the standard pH meter used (Beckman Coulter, Catalog# A58754, Brea, USA). The compensator helped adjust the electrode-offset potential. Calibration of the electrodes was done at 37°C (temperature of the mouse body) in pH buffers 4, 7 and 10. A linear Nernstian plot was obtained and was used to convert the mV readings that were obtained from the mouse wound. Measurements on every mouse wound were done at five different locations, four of which were at the periphery of the wound at 90^o^ angle and one in the center.

### Nitrate nitrite analysis

Tissues collected were weighed and introduced into eppendorf tubes with equal weights of zirconium oxide beads. Nitrite free methanol at 2 ml/g tissue was added to the tubes. Tissues were homogenized for 10 mins in a bullet blender at 4^O^C. The extracts were then centrifuged at 10000 rpm for 10 mins at 4^O^C. The methanolic supernatant was collected and analysis was performed as previously described [92].

### Lipid peroxide assay using thiobarbituric acid reactive substances

Tissue thiobarbituric acid reactive substances (TBARS) were measured by using a commercially available kit (Cell Biolabs Inc., Catalog# STA-300, San Diego, USA). Lipid peroxidation forms unstable lipid peroxides that further decompose into natural byproducts such as malondialdehyde (MDA) and 4-hydroxynonenal (4-HNE). MDA forms adducts with TBARS in a 1∶2 proportion. These aducts were measured fluorometrically at an excitation of 540 nm and emission at 590 nm. TBARS levels were then calculated in µM by comparison with a predetermined MDA standard curve.

### Isolation of DNA and 8-hydroxy-2-deoxy Guanosine (8-OH-dG) analysis

Tissue DNA was extracted by using a commercially available kit (Qiagen, Catalog# 69504, Valencia, USA). Eluted DNA was digested using nuclease P1 and the pH adjusted to 7.5–8.5 using 1 M Tris. The DNA was incubated for 30 min at 37°C with 1U of alkaline phosphatase per 100 µg of DNA and then boiled for 10 min. The 8-OH-dG DNA damage assay was performed by using a commercially available kit (Cayman Chemical, Catalog# 589320, Ann Arbor, USA). The measurements are based on a competitive enzyme immunoassay between 8-OH-dG and an 8-OH-dG-acetylcholinesterase (AChE) conjugate (8-OH-dG tracer) with a limited amount of 8-OH-dG monoclonal antibody. After conjugation, Ellman's reagent (used to quantify the number or concentration of thiol groups) was used as a developing agent and read spectrophotometrically at 412 nm. The intensity measured was proportional to the amount of 8-OH-dG that was expressed in pg/ml.

### Nitrotyrosine ELISA

Tissue nitrotyrosine levels were measured by using a commercially available kit (Cell Biolabs Inc., Catalog# STA-305 San Diego, USA). The measurements are based on a competitive enzyme immunoassay. The tissue sample or nitrated BSA were bound to an anti-nitrotyrosine antibody, followed by an HRP conjugated secondary antibody and enzyme substrate. The absorbance was measured spectrophotometrically at 412 nm and the nitrotyrosine content in the unknown sample was then determined by comparing with a standard curve that was prepared from predetermined nitrated BSA standards.

### Cell death

Tissue cell death level was measured by using Annexin V apoptosis kits (Southern Biotech, Catalog# 10010-09, Birmingham, USA) according to the manufacturer's instructions and our previously published methodology [26]. Percoll gradients were used to collect the wound cells from the homogenized wound tissue, and the cell stained with the kit reagents. Cells that lose membrane integrity allow propidium iodide to enter and bind to DNA, a phenomenon seen in case of cell death due to necrosis, whereas apoptotic cells only stain for Annexin V. The cells were then separated by FACS analysis to separate the populations staining with propidium iodide from those staining with Annexin V.

### Scanning Electron Microscopy

Tissues collected were fixed in 4% paraformaldehyde for 4 hrs at room temperature. Samples were then dehydrated in 25%, 50%, 75%, 95% and 100% ethanol for 20 min each at room temperature. Critical point drying of the tissues was performed using Critical-point-dryer Balzers CPD0202 followed by Au/Pd sputtering for 1 min in the Sputter coater Cressington 108 auto. The coated samples were attached to carbon taped aluminum stubs and were imaged using an XL30 FEG scanning electron microscope.

### *In Vivo* Imaging

Live animal images were captured using the iBox Scientia Small Animal Imaging System (UVP, LLC. Upland, CA, an Analytik Jena Company). Mice were anesthetized and placed on the imaging stage maintained at 37°C for the duration of each imaging experiment. For each time point, age-matched C57BL/6 and LIGHT^−/−^ mice was imaged using the ImageEM 1K EM-CCD (Hamamatsu, Japan), cooled to −55°C, and an optical system consisting of a 50 mm f/1.2 lens. Images were captured separately for each time point without an emission filter and at 1×1 binning. Bright field images using a white light channel were captured first at an exposure time of 150 milliseconds followed by a luminescent channel at an exposure time at 10–20 min.

### Preparation of tissue extracts

The tissues collected were prepared as previously described [21].

### Immunoblotting

Wound tissue extracts were probed for iNOS and phospho eNOS as previously described [21].

### Lipidomics

1 ml of LCMS grade ethanol containing 0.05% BHT and 10 ng of each internal standard was added to frozen wound tissues. Internal standards used were, (*d*_4_) 8-iso PGF_2α_, (*d*_11_) 5-iso PGF_2α_-VI, (*d*_4_) 6k PGF_1α_, (*d*_4_) PGF_2α_, (*d*_4_) PGE_2_, (*d*_4_) PGD_2_, (*d_4_*) LTB_4_, (d4) TXB_2_, (*d_4_*) LTC_4_, (*d_5_*) LTD_4_, (*d_5_*) LTE_4_, (*d*_8_) 5-hydroxyeicosatetranoic acid (5HETE), (*d*_8_) 15-hydroxyeicosatetranoic acid (15HETE), (*d_8_*) 14,15 epoxyeicosatrienoic acid, (*d*_8_) arachidonic Acid, and (*d5*) eicosapentaenoic acid. Samples were mixed using a bath sonicator incubated overnight at −20°C for lipid extraction. The insoluble fraction was precipitated by centrifuging at 12,000xg for 20 min and the supernatant was transferred into a new glass tube. Lipid extracts were then dried under vacuum and reconstituted in of LCMS grade 50∶50 EtOH∶dH_2_O (100 µl) for eicosanoid quantitation via UPLC ESI-MS/MS analysis. A 14 min reversed-phase LC method utilizing a Kinetex C18 column (100×2.1 mm, 1.7 µm) and a Shimadzu UPLC was used to separate the eicosanoids at a flow rate of 500 µl/min at 50°C. The column was first equilibrated with 100% Solvent A [acetonitrile∶water∶formic acid (20∶80∶0.02, v/v/v)] for two minutes and then 10 µl of sample was injected. 100% Solvent A was used for the first two minutes of elution. Solvent B [acetonitrile∶isopropanol (20∶80, v/v)] was increased in a linear gradient to 25% Solvent B to 3 min, to 30% by 6 minutes, to 55% by 6.1 min, to 70% by 10 min, and to 100% by 10.1 min. 100% Solvent B was held until 13 min, then decreased to 0% by 13.1 min and held at 0% until 14 min. The eluting eicosanoids were analyzed using a hybrid triple quadrapole linear ion trap mass analyzer (ABSciex 6500 QTRAP,) via multiple-reaction monitoring in negative-ion mode. Eicosanoids were monitored using species specific precursor → product MRM pairs. The mass spectrometer parameters were: curtain gas: 30; CAD: High; ion spray voltage: −3500 V; temperature: 300°C; Gas 1: 40; Gas 2: 60; declustering potential, collision energy, and cell exit potential were optimized per transition.

### Antioxidant inhibition and biofilm formation model

Catalase activity was inhibited by intraperitonial injection of 3-Amino-1,2,4-triazole (ATZ) at a concentration of 1 g/kg body weight 20 min prior to creating the excisional wound. GPx activity inhibition was performed by topical application of mercaptosuccinic acid at concentration of 150 mg/kg body weight immediately after wounding and the wound was covered with sterile tegaderm. 24 hrs post-wounding, 20 µl *Staphylococcus epidermidis C2* suspension at a concentration of 1×10^8^ CFU/mL was added onto the wound and this covered with sterile tegaderm. The wounds were kept moist at all times and tegaderm was replaced as soon as the sealant of the tegaderm was seen to be compromised to avoid wound contamination. All procedures were carried out in a sterile environment. The inhibitor injection protocol and application of the bacteria were repeated every week.

### Bacteria isolation and characterization

Wound exudates from LIGHT^−/−^ mice were collected using sterile cotton swabs and stored at −80°C in 1.0% w/v proteose peptone and 20.0% v/v glycerol solution until analyzed. Samples were thawed on ice, vortexed and cultured for 16–24 hrs at 37°C on tryptic soy agar plates containing 5.0% v/v defibrinated sheep blood and 0.08% w/v Congo red dye. Viable colonies were counted and then differentiated based on size, hemolytic patterns, and Congo red uptake. The cultures were examined for Grams stain reactivity and visualized using a compound light microscope. Grams negative rods were characterized using the API 20E identification kit (Biomerieux, Durham USA), grown on *Pseudomonas* Isolation Agar, oxidase activity, growth at 42°C in LB, and motility. Grams positive cocci differentiated based on catalase activity, coagulase activity, growth in 6.5% w/v NaCl tolerance test, and hemolytic activity. Biofilm production was quantified using adherence and staining of extracellular polysaccharide (slime), produced by bacteria, using Congo red staining to deduce whether or not the bacteria was a biofilm former using previously published procedures and criteria [40], [46].

### Community Minimal antibiotic inhibitory concentration assay

Community minimal inhibitory concentration (CMIC) assay was carried out with amoxicillin as described by DeLoney and Schiller [93] with the following modification. Wound exudates (containing the bacteria) were challenged with antibiotic for 12 hr with concentrations ranges from 100 to 0.78 µg/mL in tryptic soy broth after being seeded at 37oC in a humidified incubator for 4 hr prior to the assay. The CMIC is defined as the lowest antibiotic concentration that resulted in a ≤50% increase in the optical density measured at 595 nm compared to the optical density reading prior to the introduction of antibiotic.

### Tissue preparation for histology

Tissues collected were prepared as previously described [21].

### Statistical analysis

For the statistical analysis of experiments, we used Graphpad Instat Software (Graphpad, La Jolla, CA, USA) and Sigmaplot Software (SigmaPlot, San Jose, USA). Analysis of variance (ANOVA) was used to test the significance of group differences between two or more groups. In experiments with only two groups, statistical analysis was conducted using a Student's t-test.

## Supporting Information

File S1 Figure S1, Schematic illustration of oxidative and nitrosative stress cycle. Figure S2, Lactate levels, pH, and nitrosative stress are exacerbated in old LIGHT−/− mice. Figure S3, Detrimental effects of exacerbated stress on protein modification, lipid peroxidation, and DNA damage in old mice. Figure S4, Histology of normal wound healing. Figure S5, Manipulation of LIGHT−/− wounds with bacteria or individual antioxidant inhibitors does not lead to chronic wound development. Figure S6, Identification and characterization of the bioflora that colonized the old LIGHT/- chronic wounds.
(PDF)
