# Supplementary material for: A Novel Model of Chronic Wounds: Importance of Redox Imbalance and Biofilm-Forming Bacteria for Establishment of Chronicity
Source: PLoS One. 2014 Oct 14;9(10):e109848. doi: 10.1371/journal.pone.0109848 (PMC4196950; doi:10.1371/journal.pone.0109848)
Supplement: File S1 — Figure S1, Schematic illustration of oxidative and nitrosative stress cycle. Figure S2, Lactate levels, pH, and nitrosative stress are exacerbated in old LIGHT−/− mice. Figure S3, Detrimental effects of exacerbated stress on protein modification, lipid peroxidation, and DNA damage in old mice. Figure S4, Histology of normal wound healing. Figure S5, Manipulation of LIGHT−/− wounds with bacteria or individual antioxidant inhibitors does not lead to chronic wound development. Figure S6, Identification and characterization of the bioflora that colonized the old LIGHT/- chronic wounds. (PDF) [file pone.0109848.s001.pdf]

Supplementary Figures

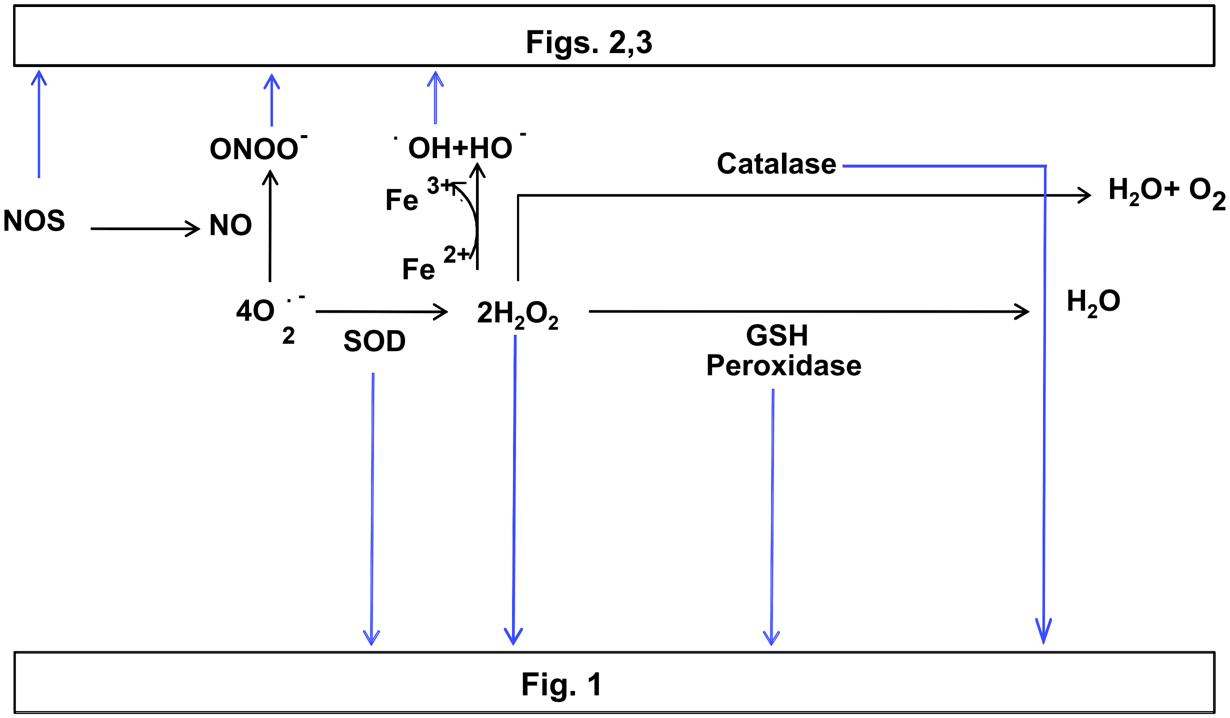

Figure S1. Schematic illustration of oxidative and nitrosative stress cycle. Superoxide dismutase (SOD) dismutates superoxide anions ($O_2^{\cdot -}$) to generate H_2O_2. The latter can be detoxified by catalase to H_2O+O_2 and by glutathione peroxidase (GPx) to H_2O. H_2O_2 can also enter the Fenton reaction in the presence of ferrous ions to give rise to ·OH+OH-. We also depict the $O_2^{\cdot -}$ interaction with nitric oxide (NO) produced by nitric oxide synthetase (NOS) to give rise to peroxinitrite anion ($ONOO^-$). The arrows directed towards Fig.1 depict the parameters involved in oxidative stress. The arrows toward Figs. 2 and 3 include the parameters of nitrosative stress and the detrimental effects of reactive species respectively (supplementary references 1,2).

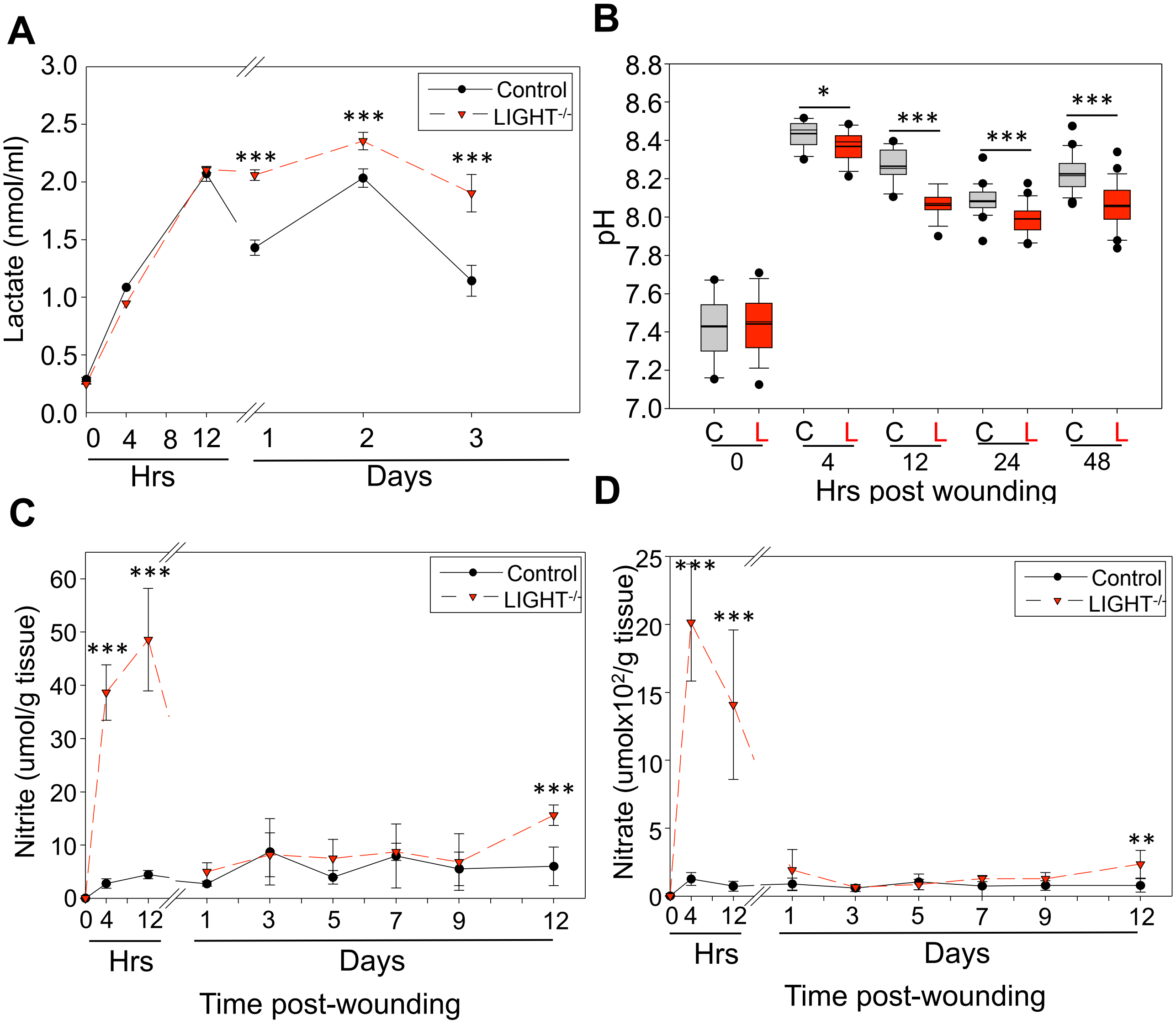

Figure S2. Lactate levels, pH, and nitrosative stress are exacerbated in old LIGHT$^{-/-}$ mice. (A) Lactate levels were measured in old LIGHT$^{-/-}$ mice during the first 48hrs of healing. Extraction of lactate was done using lactate buffer provided by the manufacturer. Oxidized intermediates reacted with a probe to give fluorescence detectable at an emission of 605nm. Data are mean ± SD, *n= 6*. (**B**) pH levels were measured using a microelectrode. A micro reference electrode was used at the same time as the pH electrode made contact with the wound tissue. Data are mean ± SD, *n= 25.* **(C,D)** Nitrite and nitrate levels were measured in old LIGHT$^{-/-}$ mice throughout the course of healing. Nitrite free methanol was used for extraction. Data shown here are representative of several independent experiments. Data are mean ± SD, *n= 8.* * *p <0.05,* ** *p <0.01,* *** *p<0.001.*

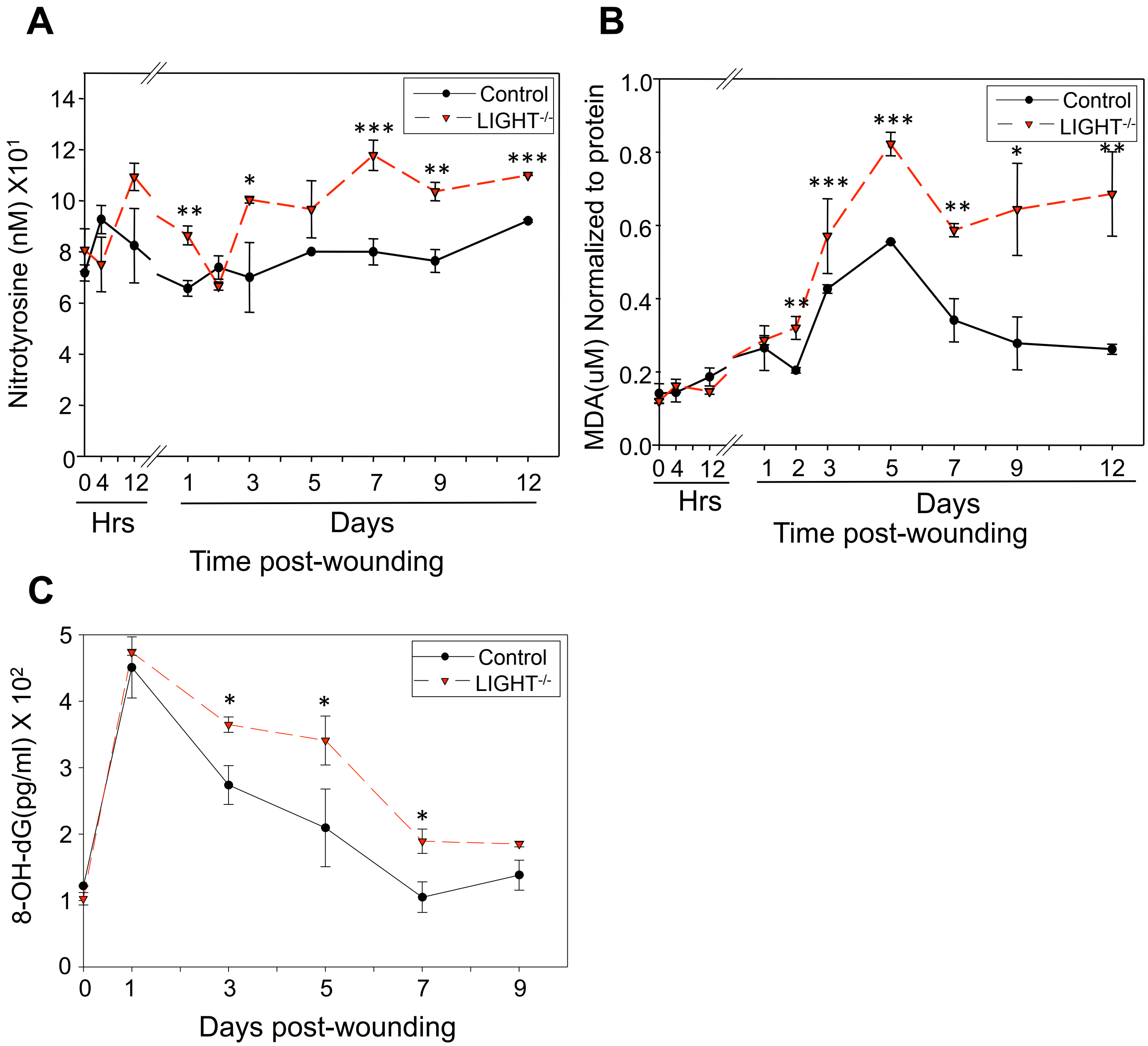

Figure S3. Detrimental effects of exacerbated stress on protein modification, lipid peroxidation, and DNA damage in old mice. (A) Nitrotyrosine levels were measured in old LIGHT-/- mice and in age matched controls using an anti-nitrotyrosine antibody and an HRP conjugated secondary antibody. Data are mean ± SD, *n=6*. **(B)** Lipid peroxidation levels were measured using thiobarbituric acid reactive substances (TBARS). The absorbance was measured spectrophotometrically at 412nm. Data are mean ± SD. *n= 6* **(C)** DNA damage was measured at various time points post-wounding by determining the levels of 8-OH-dG. Isolated DNA from the wound tissue was digested to release 8-OH-dG and measurements were based on competitive enzyme immunoassay between 8-OH-dG and an 8-OH-dG-acetylcholinesterase conjugate with a limited amount of 8-OH-dG monoclonal antibody. Data are mean ±SD. *n=4*. *p<0.05,**p<0.01,***p<0.001.

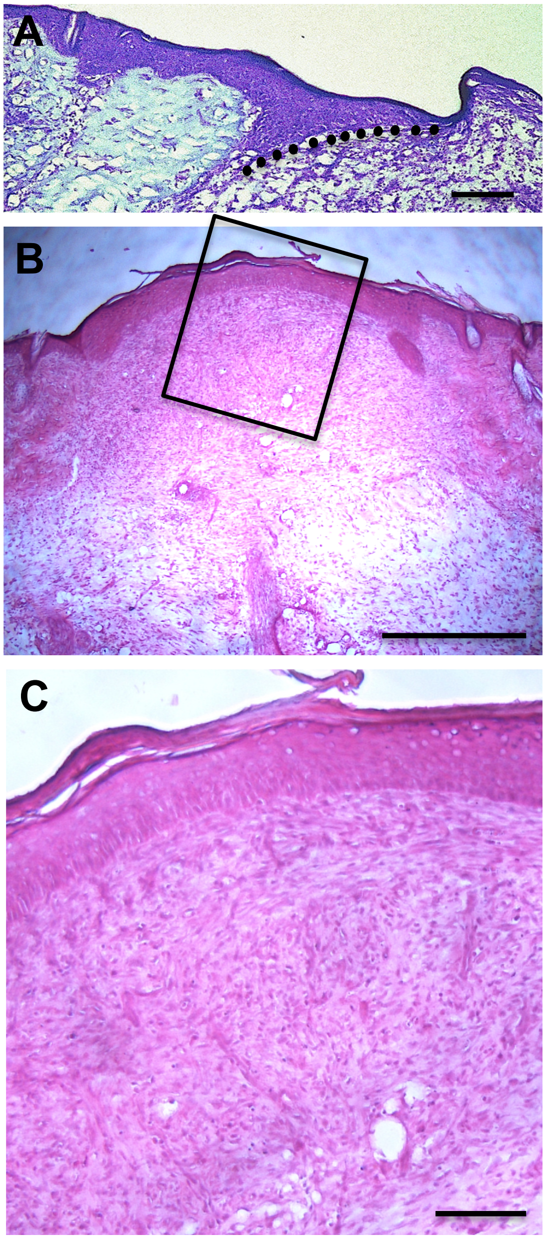

Figure S4. Histology of normal wound healing. **(A)** Migration tongue (delineated by dots) during normal wound healing. The epidermal cells form a well-defined straight migrating tongue that eventually meets the counterpart on the other side to close the wound. **(B)** Normal granulation tissue and epidermis after closure of the wound during normal healing. **(C)** Enlargement of the area depicted in the rectangle in B to show that the epidermis is well-adherent to the granulation tissue and the latter is well developed. Scale bars 50µm for (A) and (C) and 500µm for (B). Image in (A) modified from Fig. 1C of Liu et al., *BMC Cell Biology* 2009, **10**:1-15.

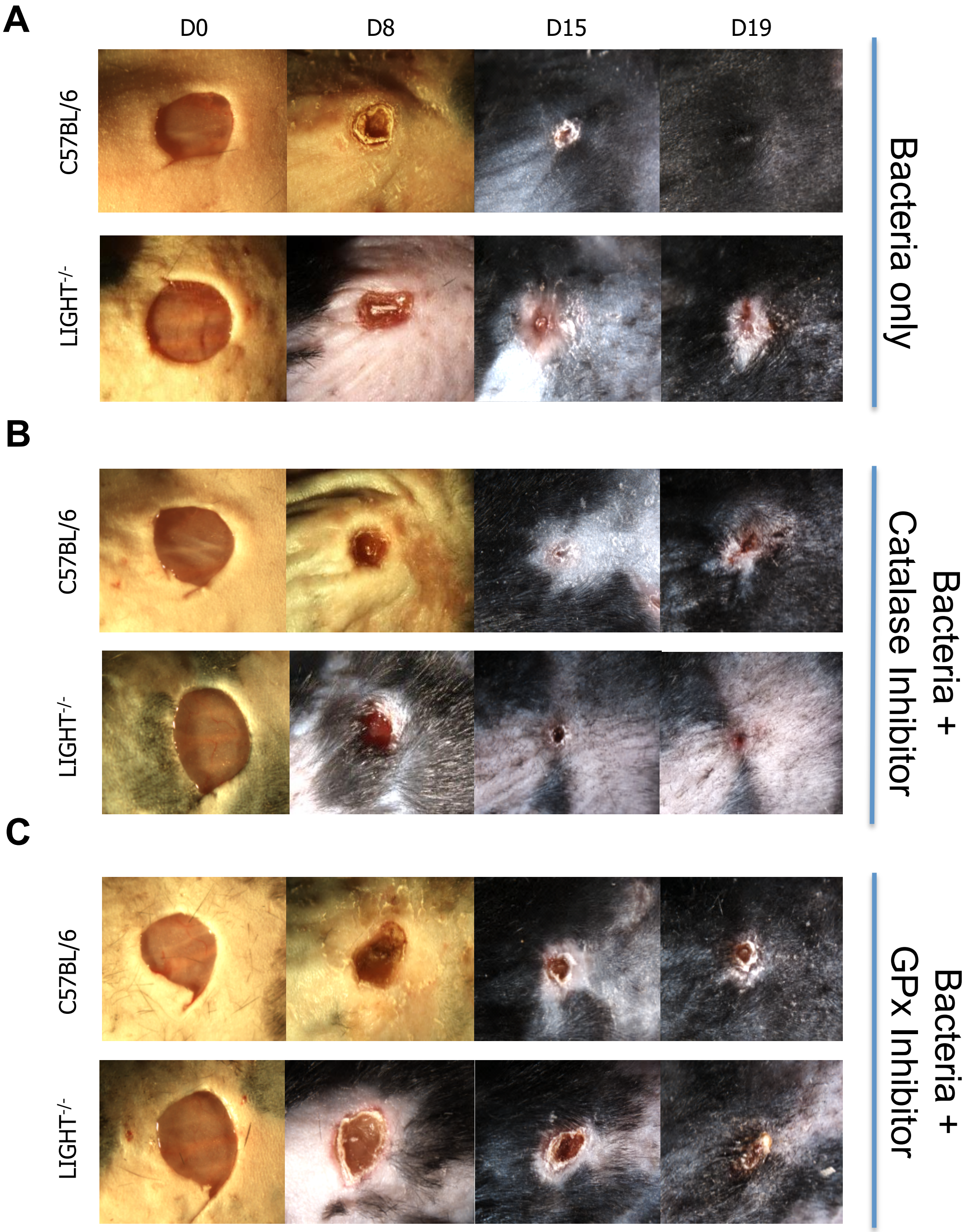

Figure S5. Manipulation of LIGHT$^{-/-}$ wounds with bacteria or individual antioxidant inhibitors does not lead to chronic wound development. (A) C57BL/6 and LIGHT$^{-/-}$ mice were topically treated with S. *epidermidis* C2 . Data are mean ± SD, *n*= *4*. **(B)** C57BL/6 and LIGHT$^{-/-}$ mice were injected with 3-Amino-1,2,4-triazole (catalase inhibitor), 20 min prior to wounding and topical application of S. *epidermidis* C2 was done 1 day post wounding. Data are mean ± SD, *n*= *4*. **(C)** C57BL/6 and LIGHT$^{-/-}$ mice were topically treated with mercaptosuccinic acid (GPx inhibitor) and topical application of S. *epidermidis* C2 was done 1 day post wounding. Data are mean ± SD, *n*= *4*. * p<0.05,** p<0.01,***p<0.001.

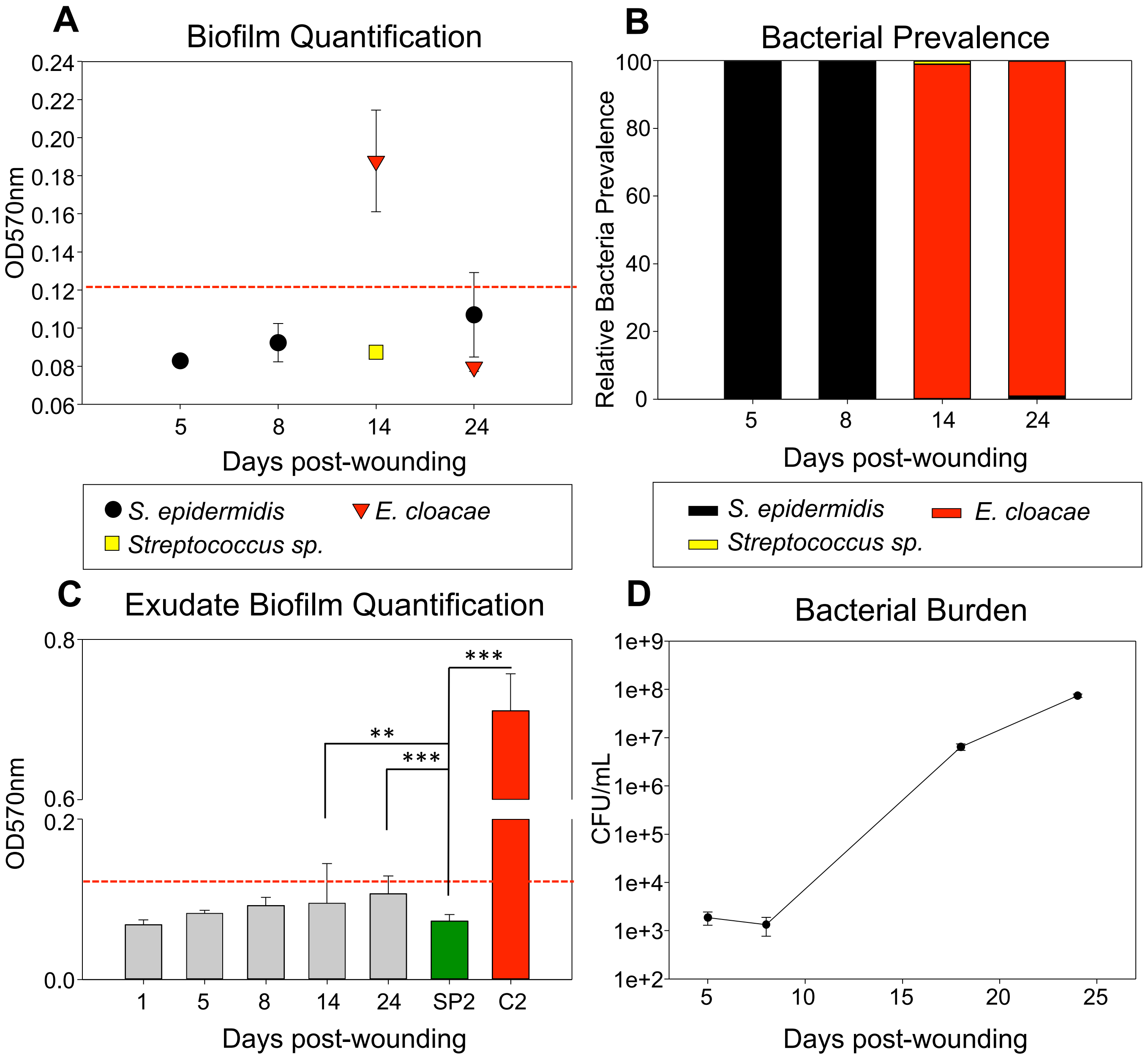

Figure S6. Identification and characterization of the bioflora that colonized the old LIGHT$^{-/-}$ chronic wounds. (A) Biofilm production/quantification of stained bacterial films adherent to plastic tissue culture plates was measured at OD570nm. Data are mean ± SD, *n*= *4*. **(B)** Bacterial identification was done by growing them on tryptic soy agar and using an identification kit. Data are mean ± SD, *n*= *4*. **(C)** Biofilm quantification of the exudates obtained from the wounds were performed at OD570nm. Biofilm quantification controls used were biofilm-negative (OD570nm < 0.125) *S. hominis* SP2 and biofilm-positive *S. epidermidis* C2. Data are mean ± SD, *n*= *4*. **(D)** Bacterial burden was evaluated based on viable colony count and differentiation. Data are mean ± SD, *n*= *4*. * p<0.05,** p<0.01,***p<0.001.
